# *Plasmodium falciparum* gene expression measured directly from tissue during human infection

**DOI:** 10.1186/s13073-014-0110-6

**Published:** 2014-11-29

**Authors:** Daria Van Tyne, Yan Tan, Johanna P Daily, Steve Kamiza, Karl Seydel, Terrie Taylor, Jill P Mesirov, Dyann F Wirth, Danny A Milner

**Affiliations:** Department of Immunology and Infectious Diseases, Harvard School of Public Health, Boston, MA USA; Broad Institute, Cambridge, MA USA; Graduate Program in Bioinformatics, Boston University, Boston, MA USA; Albert Einstein College of Medicine, Bronx, NY USA; University of Malawi College of Medicine, Blantyre, Malawi; Michigan State University, College of Osteopathic Medicine, East Lansing, MI USA; The Blantyre Malaria Project, Blantyre, Malawi; Brigham and Women’s Hospital, Boston, MA USA

## Abstract

**Background:**

During the latter half of the natural 48-h intraerythrocytic life cycle of human *Plasmodium falciparum* infection, parasites sequester deep in endothelium of tissues, away from the spleen and inaccessible to peripheral blood. These late-stage parasites may cause tissue damage and likely contribute to clinical disease, and a more complete understanding of their biology is needed. Because these life cycle stages are not easily sampled due to deep tissue sequestration, measuring *in vivo* gene expression of parasites in the trophozoite and schizont stages has been a challenge.

**Methods:**

We developed a custom nCounter® gene expression platform and used this platform to measure malaria parasite gene expression profiles *in vitro* and *in vivo*. We also used imputation to generate global transcriptional profiles and assessed differential gene expression between parasites growing *in vitro* and those recovered from malaria-infected patient tissues collected at autopsy.

**Results:**

We demonstrate, for the first time, global transcriptional expression profiles from *in vivo* malaria parasites sequestered in human tissues. We found that parasite physiology can be correlated with *in vitro* data from an existing life cycle data set, and that parasites in sequestered tissues show an expected schizont-like transcriptional profile, which is conserved across tissues from the same patient. Imputation based on 60 landmark genes generated global transcriptional profiles that were highly correlated with genome-wide expression patterns from the same samples measured by microarray. Finally, differential expression revealed a limited set of *in vivo* upregulated transcripts, which may indicate unique parasite genes involved in human clinical infections.

**Conclusions:**

Our study highlights the utility of a custom nCounter® *P. falciparum* probe set, validation of imputation within *Plasmodium* species, and documentation of *in vivo* schizont-stage expression patterns from human tissues.

**Electronic supplementary material:**

The online version of this article (doi:10.1186/s13073-014-0110-6) contains supplementary material, which is available to authorized users.

## Background

Malaria infections of humans are restricted to five species of the *Plasmodium* parasite, with high morbidity associated with three of the five species and high mortality associated predominantly with *Plasmodium falciparum*. These parasites infect over 200 million and kills more than 600,000 people each year [1]. The *P. falciparum* genome contains over 5,000 genes, yet more than half of these genes encode hypothetical proteins, expanded families of genes that interact with the immune system, and conserved proteins of unknown function [2]. The rapid generation time of *P. falciparum*, wherein one parasite divides into 16 to 24 daughter parasites within 24 to 48 h, requires an enormous devotion of energy for cell cycle replication. Additionally, this rapid development is precisely coordinated through expression states that are predictable within the *in vitro* setting over 48 h [3,4]. This predictability allows for perturbations of the parasite with drugs and other culture conditions, which can lead to global changes in gene expression [5,6].

When malaria parasite gene expression from peripheral blood is examined during human infection, there are unique physiologies present that, as yet, cannot be replicated *in vitro* [7-9]. Samples derived from human subjects are often precious, of small volume (especially in the pediatric setting), collected on media not suitable for traditional RNA analysis (for example, filter paper, formalin fixed paraffin embedded tissue) and, by definition, contaminated with human RNA. Furthermore, because parasites in the trophozoite and schizont stages sequester deep in endothelium of tissues, these parasites are only accessible by tissue biopsy or autopsy. Previous studies with autopsy tissues have measured parasite gene expression using quantitative real-time PCR (qPCR) [10-13]. Low parasite RNA abundance, poor quality RNA, and the presence of human RNA in tissue with sequestered parasites have made approaches to measure malaria gene expression in the genome-wide scale inaccessible to date.

Spanning the middle ground between low-throughput approaches like qPCR and high-throughput approaches like microarrays and RNA sequencing, the Nanostring™ nCounter® platform is a middle-throughput approach based on direct multiplex measurement of gene expression, effectively ‘counting’ transcripts using barcoded probes and automated quantification of single molecule digital imaging [14]. The nCounter® system works by directly capturing and counting individual mRNA transcripts using a target sequence bound to an immobilization bead and possessing a unique barcode. The system is capable of measuring gene expression with accuracy similar to qPCR, yet nCounter® requires significantly less preparatory work and is highly automated and capable of measuring expression in crude cell lysates. Importantly, there is no use of reverse transcription or amplification methods prior to measurement, eliminating amplification bias.

Here we develop and deploy two new techniques to measure *in vivo* gene expression of malaria parasites sequestered in patient tissues. Using a set of nCounter® malaria gene probes, we quantify malaria gene expression *in vivo*, first in a validation set of peripheral blood samples and then in a set of malaria-infected human tissue samples collected at autopsy. We also use whole-genome imputation to generate global transcriptional profiles for each sample, providing a first look at the gene expression profiles of *in vivo* sequestered malaria parasites.

## Methods

### Probe set design

The nCounter® custom code set developed for *P. falciparum* includes genes that were selected from a compendium of existing Affymetrix and two-dye array expression data as follows: the Affymetrix compendium consisted of 43 *in vivo* samples [8]; two PfSir2 knockout experiments, each with three controls [15]; 17 samples of asexual life cycle and three of sexual stages [16]; 23 samples of the gametocyte life cycle [17]; five samples of a T4 drug-treatment time course with five controls [18]; and 26 *ex vivo* patient samples [9]. The two-dye array compendium consisted of 229 drug treatment samples [5]; 208 drug treatments, environmental stresses, and substrate depletions (unpublished); and an additional 53 samples representing an *in vitro* life cycle time course experiment [4]. Genes in the nCounter® custom code set included genes that can distinguish between intraerythrocytic life cycle stages, highly differentially expressed genes from previous studies [8,9], and probes representing genes of interest expressed in gametocytes, heat shock, citric acid cycle, glycolysis, ATP binding, ubiquitin pathways, acyl CoA pathways, and others. Because of large sequence differences among parasites, we excluded highly genetically divergent gene families, such as *var* genes, in our initial probe design. A total of 328 genes were selected for the malaria nCounter® custom probe set.

nCounter® probe specificity is generated by designing two 50 bp probes that sit next to one another, yielding 100 bp total probe specificity for each gene of interest. Probe set design was carried out to maximize specificity and minimize cross-hybridization. Specifically, long direct and inverted repeats and long polynucleotide repeats were excluded, target regions were screened for cross-hybridization against the Human RefSeq mRNA database [19], and probes with stretches of more than 15 contiguous bases complementary to any non-target mRNA were excluded. Probes were also screened for inter- and intra-probe interactions, and were selected to have calculated melting temperatures (T_m_) between 78 and 83°C, with an ideal target of 80.5°C. Finally, mismatch probes were also included in the nCounter® custom probe set, as part of the negative controls.

### *In vitro* sample preparation

Serial dilutions of *in vitro* cultured 3D7 parasite lysates were prepared in Qiagen RLT buffer (Catalog #79216) with 1% beta-mercaptoethanol, and samples were stored at -80°C prior to analysis. Mock filter paper samples were prepared by spotting 200 uL of ring-stage parasitized red blood cells at 33% hematocrit onto Whatman FTA filter paper cards (GE Healthcare, Pittsburgh, PA, USA). Three punches from each card were incubated with RNA processing buffer (10 mM Tris-HCP, pH 8.0, 0.1 mM EDTA, 800 U/mL RNase OUT (Invitrogen), 200 ug/mL, 2 mM DTT) for 30 min on ice, and the supernatant was passed over a Qiagen RNeasy column (Catalog # 74104), washed with Qiagen RPE buffer (Catalog # 1018013), and eluted with nuclease-free water. Mock FFPE samples were prepared by incubating ring-stage parasitized red blood cells at 50% hematocrit with thrombin and bovine plasma in sodium citrate to induce clotting. Blood clots were fixed in 10% formalin for at least 24 h and were embedded in paraffin wax. Four slices of five microns thickness were deparaffinized using Qiagen deparaffinization solution (Catalog # 19093), followed by proteinase K digestion. Patient samples were collected previously, processed for total RNA extraction and stored at -80°C until use [9,13].

### Patient samples

All patient samples used in this study were gathered from previous studies, and their corresponding patient demographic and sample collection information is described in detail elsewhere [9,20]. The Institutional Review Boards of the University of Malawi College of Medicine, Albert Einstein College of Medicine, and the Brigham and Women’s Hospital have previously approved all aspects of the collection of these samples, including obtaining informed written consent from the parents or guardians of all patients. This research conforms to the ethical principles for medical research involving human subjects put forth by the World Medical Association Declaration of Helsinki, and to local legislation.

### Whole-genome imputation

Our imputation approach was originally developed by Donner *et al.* [21]. The Affymetrix compendium described above was curated and filtered for genes that were present in all data sets, as well as genes that vary in their expression across data sets. This filtered list of 3,696 genes was used as a training data set to select landmarks and generate the imputation model. A subset of probes, called landmarks, was selected based on their full expression profiles within the compendium. Specifically, we used the Regularized Gaussian Estimation (RGE) method proposed by Donner *et al.* [21], whereby the expression of the complete probe set is modeled as a multidimensional Gaussian set. To decide the optimal number of probes that are needed to impute the full expression profile of each sample, we computed the model fitting errors of models using between 10 and 100 probes. We found that an imputation model using 60 landmarks was ideal, since including additional probes into the model beyond 60 did not yield a large improvement in imputation accuracy.

### Differential gene expression

Imputed nCounter® gene expression for three patients with three different organs sampled was analyzed to determine parasite genes that are overexpressed *in vivo.* The three samples from each patient were compared with 11 *in vitro* microarray time points with highest correlation [4]. Because of differences across platforms, both *in vitro* and *in vivo* expression data were converted to rank-normalized values [22,23]. Differential gene expression analyses were conducted using the Limma R package [24]. Limma is a widely used R package for differential expression analyses of data arising from microarray experiments. Briefly, the method fits a linear model to the expression data for each gene, and uses an empirical Bayes approach to borrow information across genes, which is equivalent to shrinkage of the estimated sample variances towards a pooled estimate, thus making the analyses robust even when the sample size is small.

### Statistical analysis

nCounter® platform raw data were normalized with the NanoStringNorm R package [25], using the arithmetic mean to summarize negative and positive controls. We also checked the diagnostic messages from NanoStringNorm, and confirmed that all of the samples had normalization factors within the range of three standard deviations from the mean. Expression analyses were based on rank-normalized data, in order to avoid systematic variations between data sets due to differing amounts of input malaria mRNA. We used Spearman rank correlation to make comparisons between nCounter® and both Affymetrix and two-dye microarray data. To assess the relative importance of the differential gene expression results, genes were ranked by log-fold change, from largest to smallest. The top 100 overexpressed genes in each patient were compared, and 39 genes shared between all three patients are summarized in Table 1.

**Table 1:** **Summary of shared malaria genes overexpressed in three** ***in vivo*** **patient samples** ^**a**^

**Category**	**Number**	**Description** ^**b**^	**Gene names**
Gametocyte/Mosquito	10	Maximal expression during gametocyte or mosquito stages	PFA0425c, PFC0581w, PFC0755c, MAL7P1.64, MAL7P1.109, PF10_0169, PF10_0204, PF11_0163, PF13_0350, PF14_0031
Trophozoite/Schizont	10	Maximal *in vitro* expression during late trophozoite or schizont stage	PFA0210c, PFI0810c, PFI1445w, PF10_0268, PF10_0330, PF11_0048, PF11_0156, PF11_0183, PFL1565c, PF14_0366
*In vivo* expressed	7	Genes with less than 100 RPKM for any stage *in vitro*	PFC0005w, PFI1600w, PFI1830c, PF11_0203, PFL1010c, PFL1195w, PF14_0363
Ribosomal	6	Ribosomal or putative ribosomal proteins	PFC0535w, PF11_0043, PF11_0106, PF13_0171, PF13_0213, PF14_0027
Other	6	Maximal expression during ring/early trophozoite stage, or conflicting stage data	PFE1370w, PF11_0111, PF11_0224, PF14_0277, PF14_0359, PF14_0437

^a^Three parasite samples from each of three patients were compared with 11 *in vitro* microarray time points with highest correlation to each patient [4].

^b^Life cycle stages of maximal expression were assigned based on microarray and RNAseq data available from PlasmoDB.org.

RPKM: reads per kilobase per million mapped reads.

## Results

**Figure 1:**
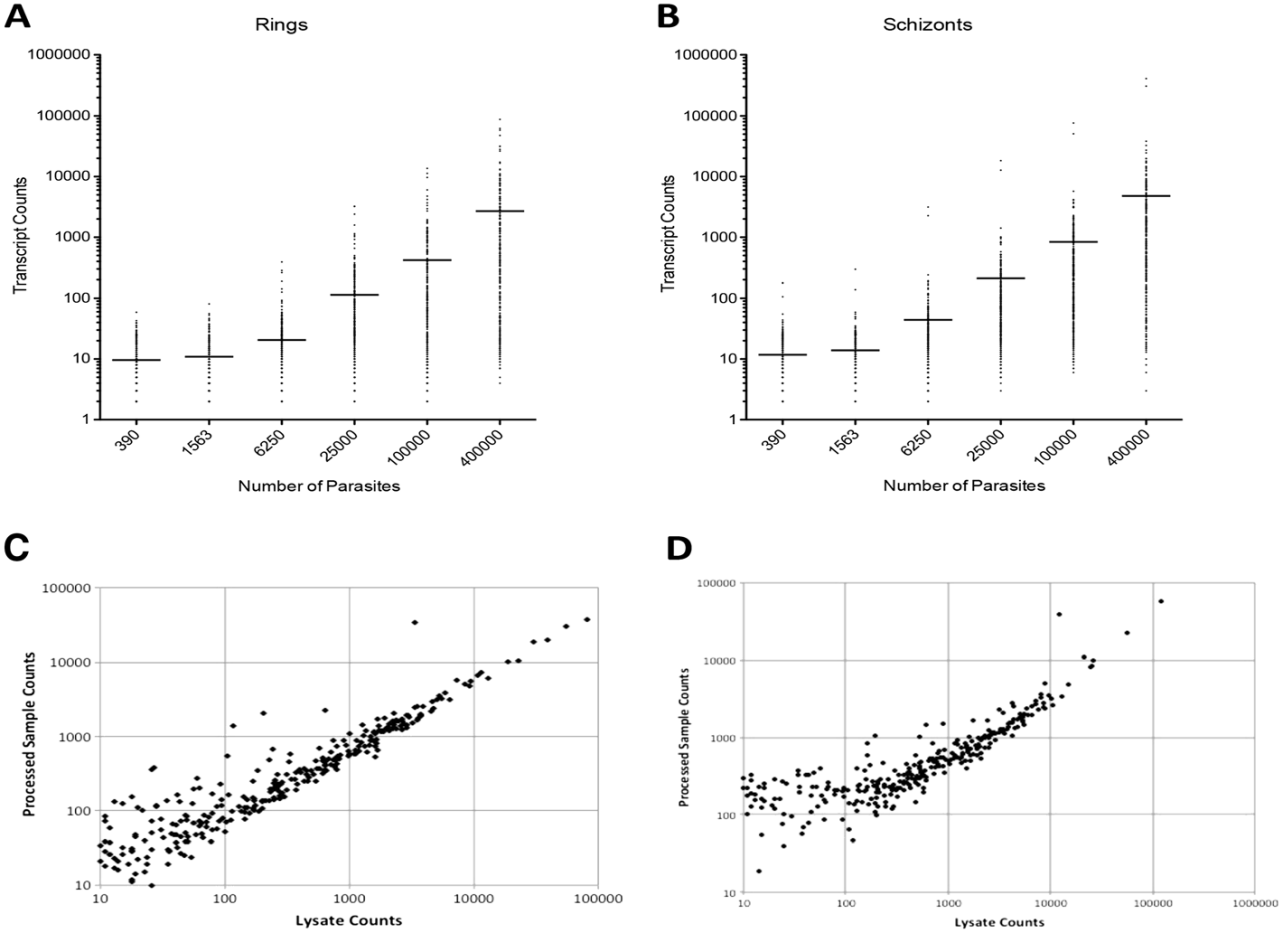
**The nCounter® platform has a large dynamic range and can be used with RNA extracted from various malaria patient sample types. (A, B)** Linearity of total transcript counts versus absolute number of *in vitro* culture-adapted 3D7 parasites that were synchronized and isolated as **(A)** rings, or **(B)** schizonts. Horizontal lines indicate mean transcript counts among all genes. **(C, D)** Processed sample transcript counts versus pre-processing lysate transcript counts for **(C)** a mock filter paper sample, and **(D)** a mock formalin fixed paraffin embedded (FFPE) sample. **(C)** Culture-adapted 3D7 parasites were synchronized and grown to ring stage, and then were spotted onto Whatman filter paper. **(D)** Culture-adapted 3D7 parasites were synchronized and grown to trophozoite stage, and then parasitized red blood cells were clotted and fixed in formalin before paraffin embedding, sectioning, and processing.

We developed a custom nCounter® malaria parasite gene probe set containing 328 genes, which were selected from a compendium of preexisting malaria expression microarray data sets (Methods). Genes selected for the probe set included genes that can distinguish between intraerythrocytic life cycle stages, highly differentially expressed genes from previous *in vivo* studies [8,9], landmark genes for imputation, and other genes of interest. This custom nCounter® probe set measured *P. falciparum* gene expression with excellent limit of detection along a 5-log dynamic range (Figure 1A and B). Transcripts were accurately counted from as few as 6,000 parasites, a volume that corresponds to 16 picoliters of parasitized red blood cells at 4% parasitemia. A range of sample types were tested and found to be suitable for analysis with nCounter®, including parasitized red blood cells spotted onto Whatman filter papers (Figure 1C), and formalin fixed paraffin embedded tissue blocks (Figure 1D). All data are deposited with NIH GEO (accession # GSE63260 and link: http://www.ncbi.nlm.nih.gov/geo/query/acc.cgi?acc=GSE63260).

**Figure 2:**
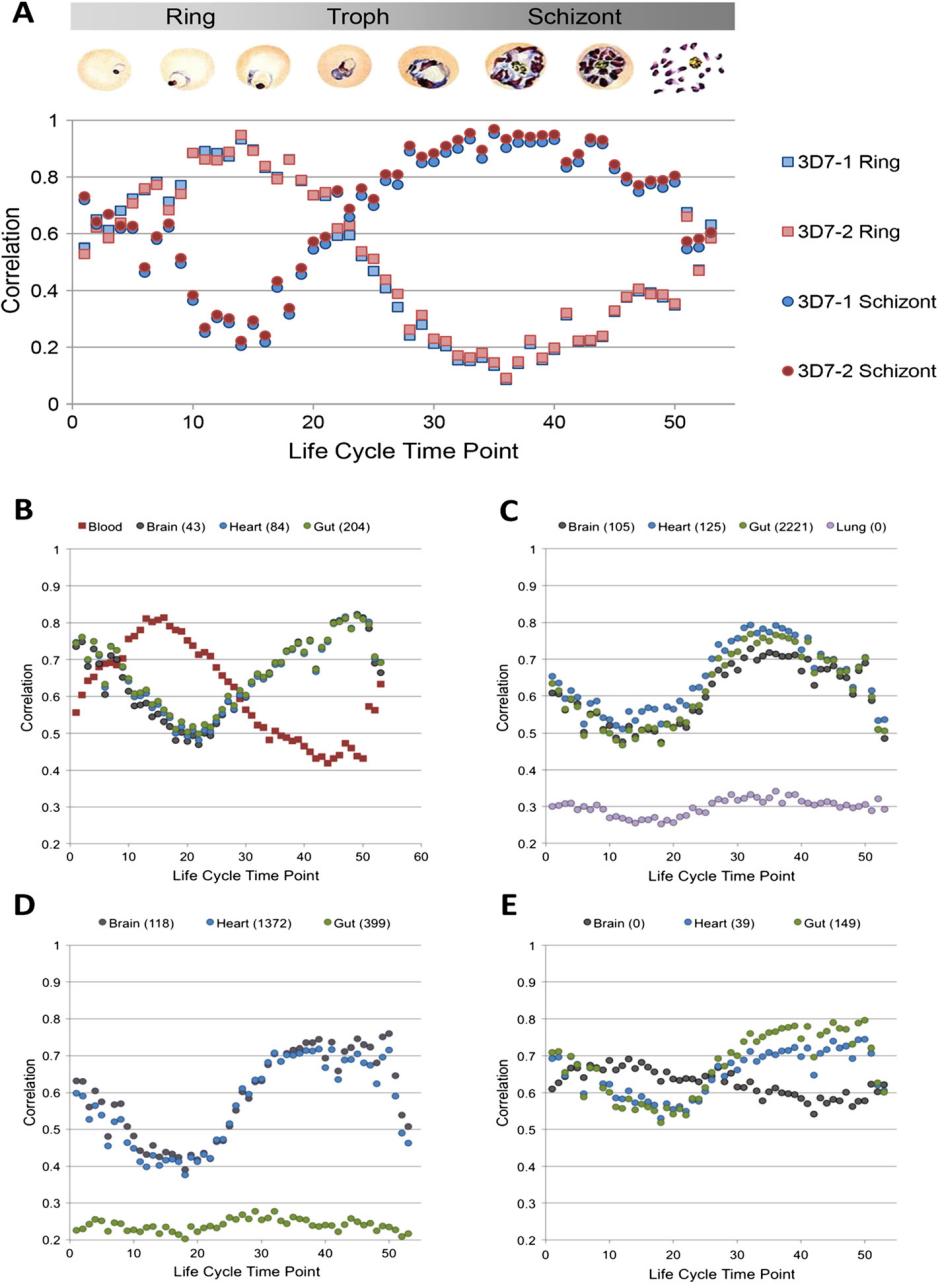
***P. falciparum*** **transcriptional profiles can be measured** ***in vitro*** **and** ***in vivo*** **with nCounter® using 328 genes. (A)** *In vitro* intraerythrocytic gene expression measured in two different 3D7 ring-stage and schizont-stage cultures. Spearman correlations between nCounter® expression and *in vitro* expression [4], is plotted versus life cycle time point. Representative illustrations of parasite developmental stages are included from [26]. **(B-E)** *In vivo* sequestered parasite gene expression measured in postmortem blood and tissues from Malawian children that succumbed to cerebral malaria **(B-D)**, or another cause of death **(E)**. Spearman correlations between nCounter® expression and *in vitro* expression are plotted versus life cycle time point. Numbers in parentheses in the legend of each panel are the number of parasites counted in 10 high-power fields by microscopy.

*In vitro* and *in vivo* parasite life cycle stages, from both peripheral blood and human tissue, are easily and accurately predicted by 328 *P. falciparum* genes measured by the nCounter® custom malaria platform (Figure 2, Additional file 1). nCounter® transcript counts were compared to a previously published *in vitro* life cycle time course, where parasite transcriptional profiles were measured every hour over the 48-h intraerythrocytic life cycle [4]. Two independent cultures of *in vitro* culture-adapted 3D7 parasites, which were synchronized and harvested at either ring stage or schizont stage, showed peak correlation with life cycle time points corresponding to the appropriate stage (Figure 2A).

The same life cycle correlation method was applied to determine parasites stages and screen peripheral blood and fresh frozen postmortem autopsy tissue samples (see Methods), collected from Malawian children who died of cerebral malaria (Figure 2B, C, D), or of another cause (Figure 2E). Parasites in peripheral blood showed the expected ring-stage profile, while parasites sequestered in tissues from the same patient showed a schizont-stage profile (Figure 2B). We looked for correlation between gene expression from these tissue-sequestered parasites and a published gametocyte expression data set [17], and observed poor correlations between tissue samples and gametocyte development profiles (data not shown), suggesting that intraerythrocytic stage profiles dominated in these samples. Life cycle correlations were observed in tissues where parasites were also seen by histology, and no correlation was seen in tissues where no parasites were found (Figure 2C). Figure 2D shows a patient in whom parasites were observed by histology in the gut tissue, but no life cycle correlation was detected using the nCounter® platform, suggesting a poor quality RNA sample. Finally, Figure 2E shows a patient that died of a cause other than cerebral malaria. In this patient, no parasites were observed by histology in the brain tissue, but the nCounter® platform detected a parasite signal with strongest correlation to ring stage parasites, suggesting that circulating ring-stage parasites were present in the brain microvasculature and were collected with the brain tissue sample.

**Figure 3:**
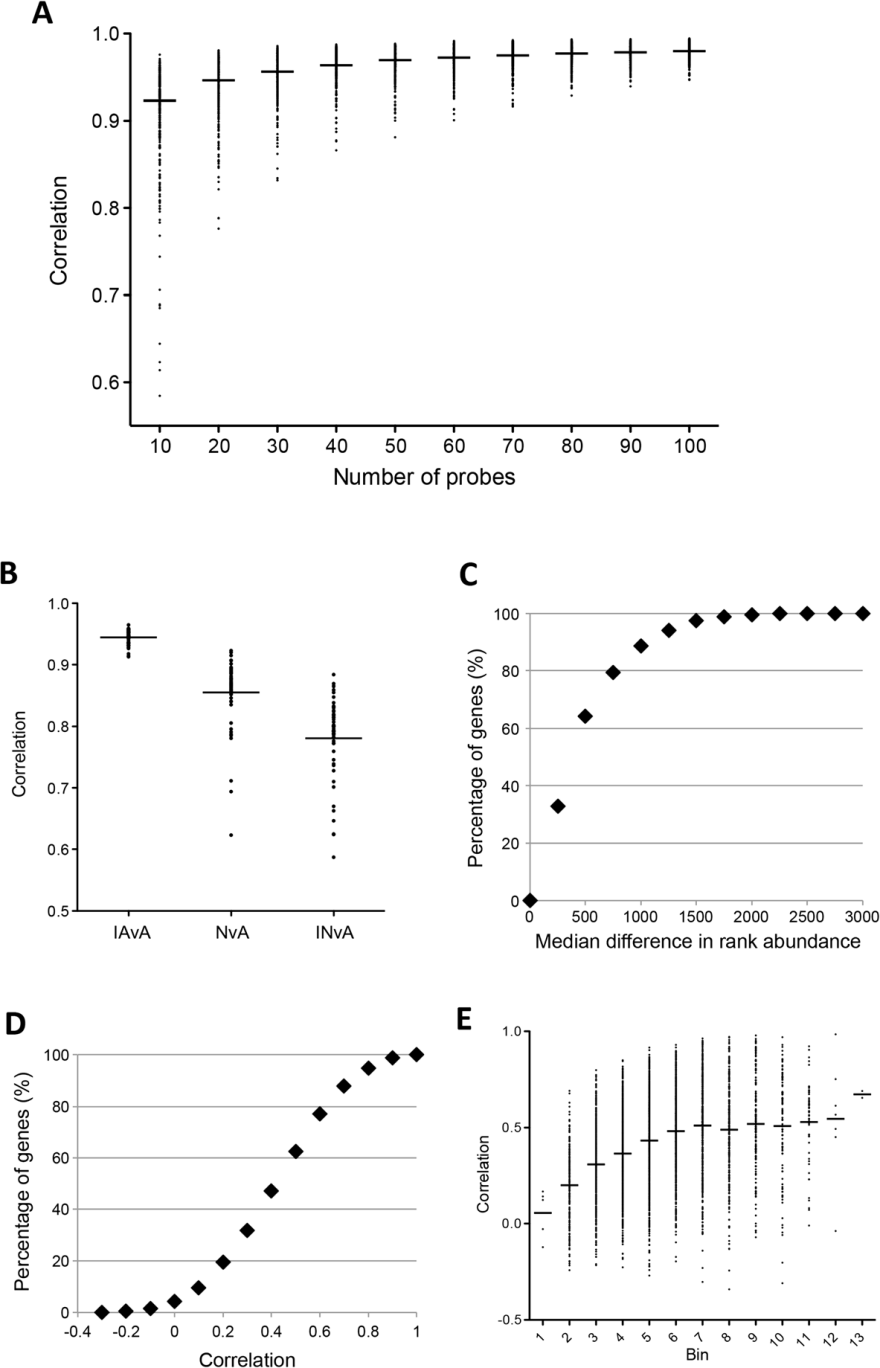
**Imputation of global expression profiles based on landmark** ***P. falciparum*** **genes. (A)** Model fitting. Spearman rank correlations between imputed and observed gene expression for 3,696 genes, based on imputation from varying numbers of probes. **(B)** Model testing. Spearman rank correlations between imputed and observed gene expression in 52 peripheral blood RNA samples, measured with both Affymetrix microarrays and nCounter®, before and after imputation. IAvA: imputed Affy vs. Affy (n = 3,969 genes); NvA: nCounter® vs. Affy (n = 328 genes); INvA: imputed nCounter® vs. Affy (n = 3,696 genes). **(C)** Cumulative distribution of median differences in rank abundance for 3,696 genes between gene expression imputed from nCounter® versus Affy, averaged over 52 peripheral blood RNA samples. **(D)** Cumulative distribution of Pearson correlations between imputed and measured gene expression, averaged over 52 peripheral blood RNA samples. **(E)** Correlation between imputed and observed gene expression scales with expression level. Pearson correlation versus quantile-normalized and log_2_-transformed gene expression for 3,696 genes averaged over 52 peripheral blood RNA samples.

We used imputation to calculate expression values for unknown genes using a small set of pre-defined genes (Methods). The imputation method was validated against parallel Affymetrix microarray data [9], and revealed that imputation based on 60 landmark genes could accurately approximate global transcriptional patterns, with minimal improvement in accuracy for imputation from more than 60 genes (Figure 3A). Imputed gene expression from 52 peripheral blood samples measured with the nCounter® platform showed good correlation with genome-wide Affymetrix microarray data gathered from the same samples (Figure 3B). To examine the accuracy of imputation on a gene-by-gene basis, we examined median differences in rank abundance (Figure 3C), and average Pearson correlation (Figure 3D), between imputed (from nCounter®) and observed (Affymetrix) expression of each gene among the 52 peripheral blood samples. Approximately 65% of imputed genes had a median difference in rank abundance lower than 500 (Figure 3C), and roughly 70% of genes had an average Pearson correlation above 0.3 (Figure 3D). Finally, the strength of correlation between imputed and observed values scaled with gene expression (Figure 3E), with more highly expressed genes having higher correlations and more lowly expressed genes having lower correlations.

**Figure 4:**
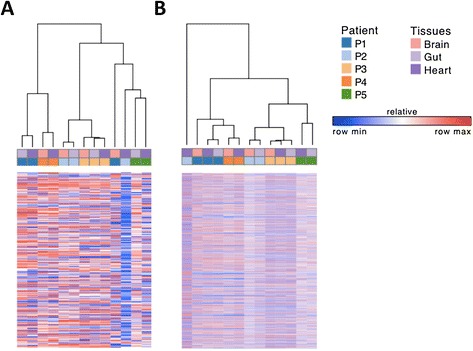
***In vivo*** **parasite gene expression clusters by patient. (A)** Hierarchical clustering of expression of 328 genes measured by nCounter® from 13 tissue samples collected at autopsy, collected from five patients and three organs. Clustering demonstrates that samples cluster by patient, rather than by organ, suggesting that parasite physiology within a patient is conserved. **(B)** Hierarchical clustering of global expression profiles imputed from nCounter® (n = 3,696 genes) shows that within-patient clusters remain intact. Note that two outliers in the first analysis (P1, Brain and P2, Heart) are further delineated as true outliers after imputation.

We used the nCounter® platform and global imputation to ask whether *in vivo* parasite samples cluster by organ or by patient (Figure 4), and to detect genes that are overexpressed by parasites sequestered in human tissues (Table 1). Hierarchical clustering of *in vivo* sequestered parasites from autopsy tissue samples showed that samples cluster by patient, rather than by organ, regardless of whether clustering is based on gene expression measured by the nCounter® platform (Figure 4A), or imputed gene expression (Figure 4B). Differential gene expression analysis between rank-normalized imputed *in vivo* and *in vitro* microarray data revealed a shared set of 39 genes that were among the top 100 overexpressed genes in all three of the analyzed patients (Table 1). This set of *in vivo* overexpressed genes included 10 genes with maximal expression during sexual and mosquito stages, 10 genes with maximal *in vitro* expression during trophozoite and schizont stages, seven genes that are not expressed *in vitro*, and six genes that code for ribosomal proteins. These data indicate that parasites sequestered in human tissues may exist in a unique physiological state.

## Discussion

The development of new tools and adaptation of existing tools for use by malaria researchers and clinicians to meet elimination and eradication goals is a priority. Ideal tools should give maximal information from minimal amounts of biological sample, and be both affordable and easy to use. Our goal is to develop and deploy such tools for global use by the malaria community. Here we show how adaptation of two existing tools - the nCounter® platform and imputation of global gene expression - can be leveraged to uncover the expression profiles of *in vivo* sequestered malaria parasites. These unique transcriptional profiles have, until now, been impossible to measure on a global scale.

Although sequencing and array technologies are sensitive and thorough, deployment of such techniques to field settings, contamination of human mRNA, and/or cost inefficiency of these tools as screening methods make their usefulness limited for immediate screening and characterization of large numbers of samples. By measuring just 328 parasite genes, we were able to distinguish between asexual life cycle stages using picoliter volumes of infected red blood cells. Moreover, we could classify parasite physiological states quickly and as accurately as measurement with microarrays. Thus, the nCounter® platform and our custom *P. falciparum* probe set could be a valuable screening method to assess RNA quality in crude lysate samples and determine parasite life cycle stages, both of which are useful to identify meaningful groups of samples for more costly and labor intensive experiments, such as RNAseq.

Because both human [21] and malaria parasite [7-9] physiologic expression states are predictable, they can be accurately determined using subsets of carefully selected landmark genes. Full-scale prediction of global expression profiles using established imputation methods allowed us to query the expression of nearly 3,700 genes from just 60 landmarks. When we compared imputed expression profiles with expression data from the same RNA samples measured with microarrays, we found that correlations between imputed and observed expression were only slightly lower than cross-platform correlations performed without imputation (Figure 3B). Nonetheless, genes that were lowly expressed in all samples showed poor correlations between imputed and observed expression across the 52 samples we used to test the imputation model (Figure 3E). For this reason, imputation is intended as a screening approach, which can be utilized in picking samples for more comprehensive experiments such as RNAseq. Data from further studies of parasite physiology will be useful for refining the imputation model, to incorporate additional variation and more accurately impute lowly expressed genes.

Previous studies have applied nCounter® to other infectious diseases [27-29], however this is the first application to measure malaria parasite physiological states *in vivo*. We found that parasites sequestered in human tissue overexpress genes that are enriched during gametocyte and mosquito stages, as well as genes that are not normally expressed *in vitro*. We also found that sample clustered largely by patient, with differences between samples from the same patient likely due to different time points of sample collection. Although it is possible that differences in genetic makeup of parasites in an organ could result in different expression patterns within the same patient, our previous data have shown that genotypes are largely conserved across organs [30]. Examination by gene set enrichment analysis (GSEA) of our imputed samples also revealed overexpression of gametocyte genes, but these findings were not statistically significant (data not shown). Importantly, many of the shared overexpressed *in vivo* genes encode conserved proteins of unknown function, indicating unexplored areas of parasite physiology that are relevant *in vivo*. Using additional data sets and collaborations with others in the malaria community, future custom nCounter® arrays can be designed to study specific gene sets, at costs comparable to qPCR but with massive savings of both time and throughput.

## Conclusions

In this study, we used a custom *P. falciparum* nCounter® probe set to measure malaria parasite gene expression in a variety of samples derived from both *in vitro* experiments and *in vivo* human infections, using very small amounts of mRNA. We used newly developed imputation methods to predict the global expression profiles of samples by measuring only a subset of genes. We applied these methods to study the *in vivo* transcriptional profiles of parasites sequestered within the tissues of Malawian children who succumbed to cerebral malaria. For the first time, we measured the expression profiles of sequestered schizont-stage parasites within a human infection. The nCounter® platform is of great utility to the malaria community, as it is amenable to many different types of samples, and imputation allows researchers to measure a small number of genes and gain an understanding of global expression patterns.

## Additional file

Additional file 1
**Lists processed (normalized and log2-transformed) nCounter® data for all** ***in vivo*** **patient samples presented.**
